# De-scattering Deep Neural Network Enables Fast Imaging of Spines through Scattering Media by Temporal Focusing Microscopy

**DOI:** 10.21203/rs.3.rs-2410214/v1

**Published:** 2023-06-08

**Authors:** Zhun Wei, Josiah R. Boivin, Yi Xue, Kendyll Burnell, Navodini Wijethilake, Xudong Chen, Peter T. C. So, Elly Nedivi, Dushan N. Wadduwage

**Affiliations:** 1 Center for Advanced Imaging, Faculty of Arts and Sciences, Harvard University, Cambridge, MA 02138, USA.; 2 State Key Laboratory of Modern Optical Instrumentation, ZJU-Hangzhou Global Science and Technology Innovation Center, College of Information Science and Electronic Engineering, Zhejiang University, Hangzhou 310027, China.; 3 Picower Institute for Learning and Memory, Massachusetts Institute of Technology, Cambridge, MA 02139, USA.; 4 Dept. of Electrical Engineering and Computer Sciences, University of California, Berkeley, CA 94720, USA.; 5 Department of Electrical and Computer Engineering, National University of Singapore, 4 Engineering Drive 3, Singapore 117583, Singapore.; 6 Dept. of Biological Engineering, Massachusetts Institute of Technology, 77 Massachusetts Ave., Cambridge, MA 02139, USA.; 7 Laser Biomedical Research Center, Massachusetts Institute of Technology, 77 Massachusetts Ave., Cambridge, MA 02139, USA.; 8 Dept. of Mechanical Engineering, Massachusetts Institute of Technology, 77 Massachusetts Ave., Cambridge, MA 02139, USA.; 9 Department of Brain and Cognitive Sciences, Massachusetts Institute of Technology, Cambridge, MA 02139, USA.; 10 Department of Biology, Massachusetts Institute of Technology, Cambridge, MA 02139, USA.

## Abstract

Today the gold standard for *in vivo* imaging through scattering tissue is point-scanning two-photon microscopy (PSTPM). Especially in neuroscience, PSTPM is widely used for deep-tissue imaging in the brain. However, due to sequential scanning, PSTPM is slow. Temporal focusing microscopy (TFM), on the other hand, focuses femtosecond pulsed laser light temporally while keeping wide-field illumination, and is consequently much faster. However, due to the use of a camera detector, TFM suffers from the scattering of emission photons. As a result, TFM produces images of poor quality, obscuring fluorescent signals from small structures such as dendritic spines. In this work, we present a de-scattering deep neural network (DeScatterNet) to improve the quality of TFM images. Using a 3D convolutional neural network (CNN) we build a map from TFM to PSTPM modalities, to enable fast TFM imaging while maintaining high image quality through scattering media. We demonstrate this approach for *in vivo* imaging of dendritic spines on pyramidal neurons in the mouse visual cortex. We quantitatively show that our trained network rapidly outputs images that recover biologically relevant features previously buried in the scattered fluorescence in the TFM images. *In vivo* imaging that combines TFM and the proposed neural network is one to two orders of magnitude faster than PSTPM but retains the high quality necessary to analyze small fluorescent structures. The proposed approach could also be beneficial for improving the performance of many speed-demanding deep-tissue imaging applications, such as *in vivo* voltage imaging.

## Introduction

Imaging large 3D volumes at high spatial and temporal resolution is a fundamental challenge for *in vivo* microscopy. With the inherent advantages of high spatial resolution, low phototoxicity, and deep penetration, point-scanning two-photon microscopy (PSTPM) is currently the gold standard for *in vivo* imaging of fluorescently labelled structures^1-4^ . However, due to sequential point scanning, PSTPM is relatively slow, limiting the size, resolution, or the speed of volumetric imaging ^5,6^.

Temporal focusing microscopy (TFM)^7-9^ overcomes the speed limitation of PSTPM by wide-field excitation and camera-based acquisition. Instead of focusing in space, TFM controls pulse dispersion to ensure that femtosecond pulse width occurs only at the focal plane. This approach enables depth selective two-photon excitation of millions of pixels simultaneously. While TFM excitation photons can penetrate through scattering tissue due to their long wavelengths, shorter-wavelength emission photons suffer from substantial scattering on their way to the detector; therefore, in a wide-field setting, some photons are mapped to incorrect detector pixels. In contrast, PSTPM integrates all the emission photons and assigns all the signal to the known scan locations; TFM is thus much more sensitive to tissue scattering. The scattered photons degrade the image quality, hindering TFM’s ability to image small, dim fluorescent structures. Therefore, while TFM has been used successfully for *in vivo* imaging of large structures such as neuronal cell bodies at relatively low resolution^10,11^, its use has not been demonstrated for *in vivo* imaging of small structures such as dendritic spines, whose relatively dim fluorescence is often lost to scattering. Overcoming the scattering limitation of TFM would open the door to a wealth of experiments that are infeasible with traditional PSTPM and TFM.

In most imaging modalities, such as TFM, the imaging process can be treated as a forward model (*f*) that transforms the ground truth image (*x*) to the observed image (*y*), where *y* is usually degraded by noise contamination, scattering effects, low-pass filtering, and subsampling^12^. Reconstructing *x* from the observed *y* is a challenging, ill-posed inverse problem (*f* ^−1^) since the forward model could map multiple different images to the same observation image^13^. Traditionally, these ill-posed inverse problems are solved using slow iterative algorithms by considering prior information with regularizations^14^. However, the accuracy of these model-based algorithms is contingent upon successfully capturing the right prior information, a challenging task for complex image structures in many practical microscopy applications.

To incorporate priors for challenging inverse problems, machine learning has proven extremely capable, especially for vision tasks^12,15-17^. Recently, thanks to increased computational power and accumulated data, significant progress has been made in the field of machine learning, where a much deeper network can be used to achieve state-of-the-art performance for many tasks. For example, deep learning has shown its success in image classification^18,19^, prediction^20^, segmentation^21,22^, denoising^23^, and other linear or nonlinear inverse problems^16,24-26^. In microscopy, it is becoming an increasingly important tool for image reconstruction^12^, image restoration^15^, deconvolution^27,28^, super-resolution^29-31^, axial-resolution enhancement^32^, and other tasks that are challenging or complicated using traditional techniques^33^. It has been shown that, provided with sufficient training examples, deep learning is capable of extracting useful information^34^ and recovering high-frequency information from raw data^35^, which can be understood as a universal approximation^36^. However, to the best of our knowledge, deep learning based inverse solvers have not been demonstrated for imaging deep through scattering tissue assisted by a physical forward-solver.

In this work, we first model the forward scattering process (*f*) with a scattering point-spread-function that gradually changes with Z-depth^2^. We map a large library of domain specific PSTPM volumes through *f* to their corresponding TFM volumes with the forward solver. We then train DeScatterNet to learn a volumetric inverse map, *f*^−1^, from TFM to PSTPM images. Our DeScatterNet addresses the problem of low image quality in TFM. Rather than improving the performance using a mechanical approach, such as HiLL^37^, mosTFM^38^, TRAFIX^39^ or DEEP-TFM^40^ microscopy, we utilize a data-driven approach to recover meaningful patterns from high-speed TFM measurements. The results are verified by both numerical and *in vivo* experiments. Our results show that the deep learning approach can recover biologically meaningful features that were unrecognizable in the TFM images, including dendritic spines. By recovering small features that would otherwise be lost to scattering in TFM images, our deep learning approach enables rapid imaging of large 3D volumes while achieving high image quality, nearly 50 times faster than PSTPM.

## Results

### Numerical results:

**Fig. 1:**
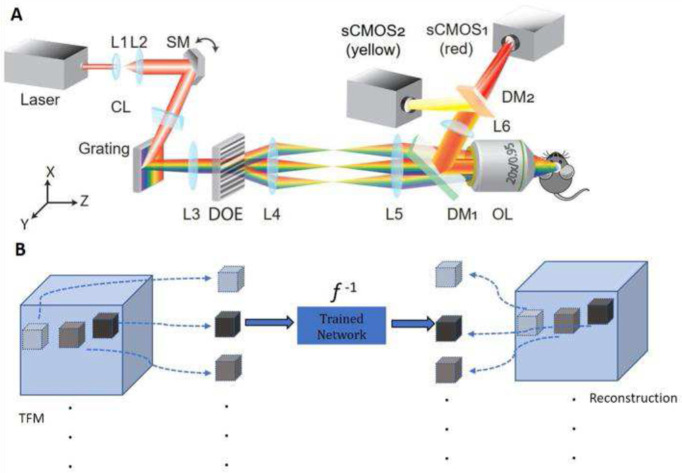
The optical schematic of the TFM setup (A): SM: scanning mirror. CL: cylindrical lens. Grating: dispersion along the x-axis. L3, L4: relay lenses. DOE: diffractive optical elements, to generate multiple lines for parallel scanning. L5, L6: tube lenses. DM: dichroic mirror. OL: objective lens. DeScatterNet to solve the inverse problem *f*^−1^ (B): During the test stage, cubes generated from TFM image stacks with a 64 μm range in z-depth are used as input, where the outputs are benchmarked with the ‘ground truth’, i.e., PSTPM images.

To build the DeScatterNet model with PSTPM data, we synthetically generated TFM cubes from 33 PSTPM volumes that each included the dendritic arbor of a layer 2/3 pyramidal neuron imaged *in vivo* in the mouse brain (See Methods). In the training stage, for each PSTPM volume, regions of interest were randomly selected to synthetically generate hundreds of smaller TFM cubes for training purposes (See Methods). As presented in Fig. 1B, during the test stage, cubes generated from TFM image stacks are used as input, where the outputs are benchmarked with the ‘ground truth’, i.e., PSTPM images.

**Fig. 2.**
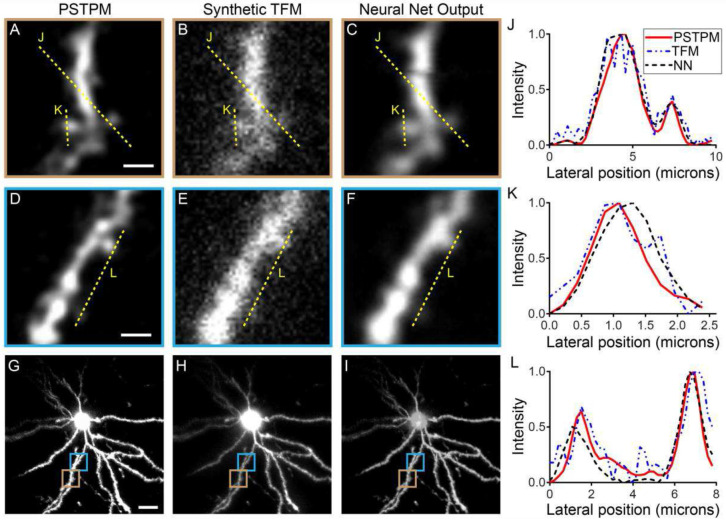
Numerical Results. Representative images of the dendrites of a neuron whose synthetic TFM image was used to test the performance of the neural network. Enlarged views of two example branches are shown in A-C and D-F, with maximum intensity Z projections of the full field of view shown in G-I. The neuron’s dendritic arbor was imaged *in vivo* using PSTPM (A, D, G). TFM images (B, E, H) were synthetically generated from PSTPM stacks and used as inputs to the neural network. The neural network (C, F, I) was then able to extract relevant features from the synthetic TFM image (B, E, H), showing good correspondence with the ‘ground truth’ PSTPM image (A, D, G). The locations of the example branches are indicated by boxes on the maximum intensity Z projections shown in G-I. Quantitative comparisons among the PSTPM, TFM, and neural network output (neural net abbreviated as NN) are shown, where the normalized intensity profiles varying with the lateral positions are presented (J, K, L). The fluorescence intensity quantification shows clearly defined peaks at the sites of dendritic spines in the PSTPM and neural net images, while TFM images are hazy and show additional small peaks at incorrect positions. Scale bars: 2 μm for A-F, 20 μm for G-I.

Fig. 2 shows representative results of reconstructing dendrites of a neuron using the proposed deep learning model. In this example, the TFM inputs to the network were synthetically generated from a cell imaged with PSTPM; neither during training nor during validation had the network seen this cell. The proposed learning model reconstructs some important small features that were either missing or hardly recognized in TFM results (Fig 2B, E, and H). In Figs. 2J, K, and L, we also present the quantitative results by comparing the normalized intensity profiles in the lateral direction. The normalized intensity shows clearly defined peaks at the sites of dendritic spines in the PSTPM and neural net output images. However, in TFM images, these peaks are either obscured by scattered fluorescence along the dendritic shaft or distorted into multiple peaks (in Figs. 2J-L). The proposed method was further validated by testing on synthetic blood vessel data, showing that it works for other types of *in vivo* imaging data (Supplementary Section 5).

### Experimental results, dendritic spine analysis:

**Fig. 3.**
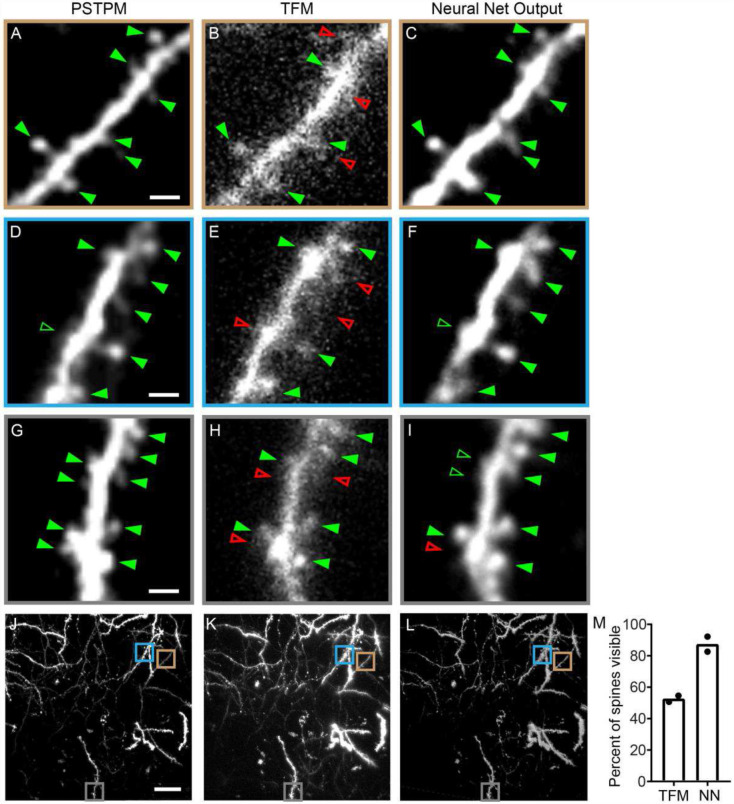
Spine scoring on experimental data. Representative images of the apical dendrites of a neuron whose TFM image was used to test the performance of the neural network. Enlarged view of three example branches located at depths of 66 μm (A-C), 41 μm (D-F), and 64 μm (G-I) from the surface of the brain. The neuron’s apical dendritic tuft was imaged *in vivo* using PSTPM (A, D, G) and TFM (B, E, H). The neural network (C, F, I) was then able to extract relevant features from the TFM image (B, E, H), showing good correspondence with the ‘ground truth’ PSTPM image (A, D, G). Green arrows denote dendritic spines scored on the PSTPM image (A, D, G). Each spine was then scored as present (green arrow) or absent (red open arrow) in the TFM (B, E, H) and neural net (C, F, I) images. Spines that are out of focus in the Z plane used for the example image are denoted by open green rectangles. Maximum intensity Z projection of the full 3D image stack is shown for the PSTPM (J), TFM (K), and neural net (L) images, with the example dendrites shown in A-I marked by color-coded boxes. Two human scorers analyzed the images. Of the 270 (scorer 1) or 271 (scorer 2) spines marked as present in the PSTPM image, 51% (scorer 1) or 55% (scorer 2) were visible in the TFM image, while 92% (scorer 1) or 83% (scorer 2) were visible in the neural net output image (M, neural net abbreviated as NN). Scale bars: 2 μm for A-I, 20 μm for J-L.

We next validated our method using experimental data. The apical dendritic tuft of a layer 2/3 pyramidal neuron in the mouse visual cortex was imaged *in vivo* using TFM. The same neuron was then imaged using PSTPM. TFM images served as the input for the trained network, while PSTPM images of the same dendrites served as ‘ground truth’ data to benchmark network performance. The TFM 3D image stack was divided into cubes and fed into the trained network. The reconstructed results generated by the trained network were then compared to the ground truth PSTPM image as shown in Fig. 3 for a neuron expressing mScarlet-I as a cell fill.

We quantified the ability of the trained network to reconstruct important features of the neuron by scoring dendritic spines across the full image stack. The image stack spanned a 200 by 200 μm field of view in XY and a 64 μm range in Z (depth: 14-78 μm from the surface of the brain), showing the fluorescently labeled apical dendritic tuft of the layer 2/3 pyramidal neuron with its resident spines. Dendritic spines were scored using established criteria based on a common multi-lab consensus^4,5^ (see Methods). A representative dendritic branch at a depth of 66 μm from the surface of the brain is shown for the PSTPM image (Fig. 3A), TFM image (Fig. 3B), and neural net output image (Fig. 3C). Spines scored on the PSTPM image are denoted by green triangles in Fig. 3A. Each of these spines is then marked as present (filled green triangle) or absent (open red triangle) in the TFM and neural net output images (Fig. 3B-C). Two additional example branches are shown in Fig. 3D-F and Fig. 3G-I, with spines marked in the same manner. Open green triangles mark the locations of spines that are out-of-focus in the Z plane used for the example image (further illustrated in Supplementary Figure 6). A maximum intensity Z projection of the full image stack is shown for the PSTPM image (Fig. 3J), TFM image (Fig. 3K), and neural net output image (Fig. 3L), with the location of each example branch indicated by color-coded rectangles. Results of the spine analysis are shown in Fig. 3M. Our results indicate that the trained neural network can reconstruct dendritic spines that were previously lost in the scattered fluorescence of a TFM image, producing an image that shows close correspondence with the ground truth PSTPM image.

To determine whether our method can generalize to different emission wavelengths, which have different scattering lengths, we re-trained the network and tested our method on a cell expressing eYFP rather than the mScarlet-I label (Supplementary Figure 6). The eYFP-expressing neuron was imaged *in vivo* using PSTPM and TFM. The PSTPM 3D image stack served as ‘ground truth’ data, while the TFM 3D image stack was divided into cubes and fed into the trained network. The reconstructed results generated by the trained network were then compared to the ground truth PSTPM image as shown in Supplementary Figure 6. Our results indicate that the trained network successfully recovered spines lost to scattering in the TFM image from the eYFP-expressing neuron, suggesting that our method generalizes well across multiple emission wavelengths.

The above analysis was designed to search for “false-negative” spines, i.e. spines that were visible in the PSTPM image but not visible in the TFM and/or neural net output images. To check for “false-positive” spines, we searched for spines present in the neural net output images but not in the PSTPM images for a randomly selected subset of dendrite branches. Our false positive rate was 4.5% (i.e. 7 false positive spines detected out of 154 total spines).

### Experimental results, fluorescence intensity quantification:

**Fig. 4.**
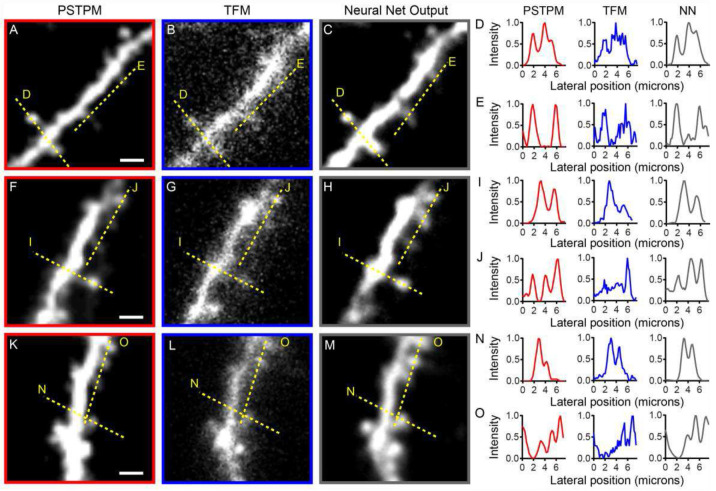
Fluorescence intensity measurements for PSTPM, TFM and neural net (NN) output images. Images are the same as those shown in Fig. 3, with each row of images showing a segment of dendrite imaged using PSTPM (A, F, K) and TFM (B, G, L), then reconstructed from the TFM image using the artificial neural network (C, H, M). Fluorescence intensity was measured along the yellow dashed lines shown on the images, with traces of normalized intensity shown in the right panel. Scale bars, 2 μm.

We further characterized our experimental results by quantifying the fluorescence intensity along regions of interest (ROIs) that included dendritic spines and the dendritic shaft (Fig. 4). In accordance with the spine analysis shown in Fig. 3, the fluorescence intensity quantification shows clearly defined peaks at the sites of dendritic spines in the PSTPM and neural net output images. These peaks are often obscured by surrounding noise from scattered fluorescence in the TFM images. For example, the PSTPM and neural net output traces in Fig. 4J show three peaks representing three dendritic spines, while the TFM trace shows only the brightest of these three spines; the dimmer two spines are obscured by scattered fluorescence along the dendritic shaft in the TFM trace (Fig. 4J). Similar results are shown for the eYFP-expressing neuron in Supplementary Figure 7. For example, in Supplementary Figure 7E and N, spine heads show small peaks that are clearly distinguishable from the larger dendritic shaft peak in the PSTPM and neural net output images, but are indistinguishable from the haze surrounding the dendritic shaft in the TFM image. These results suggest that the trained network can recover features lost to scattering in TFM images across multiple emission wavelengths.

### Structure similarity (SSIM) values quantification, and comparison to other methods:

To quantitatively evaluate the performance of the proposed method, we calculated and compared Structure similarity (SSIM) values of the experimental results from PSTPM, TFM, and neural net output. Specifically, 12 dendrite branches from the experimental results were cropped and their SSIMs were calculated (See Supplementary Section 3 for details). Our results clearly demonstrate that the proposed neural net method has a consistent SSIM advantage over TFM.

In addition, we compared the proposed method with four other networks and one traditional deconvolution algorithm called the Generalized Tikhonov algorithm^41,42^. As shown in Supplementary Figure 4, the output of the proposed neural net provides the best correspondence with ground truth PSTPM data, as compared to these alternative methods.

## Discussion and Summary

Our results indicate that the presented DeScatterNet model complements high-quality TFM imaging of large 3D volumes through scattering media. The network was capable of recovering small, biologically relevant structures that would be lost in traditional TFM due to scattering and low image quality. Using both simulation and experimental data, we show that our trained neural network can reconstruct dendritic spines from TFM images of layer 2/3 pyramidal neurons in the mouse visual cortex. Using PSTPM images of the same dendrites as ground truth to test the performance of our trained network, we determine that our trained network recovers the vast majority of the spines present in the PSTPM data, while only half of those spines are detectable in the TFM data (Fig. 3, Supplementary Fig. 6). Further quantification of fluorescence intensity shows that the trained network produces clear peaks in fluorescence at the sites of dendritic spines, similar to those present in the PSTPM image, while those same spines are often buried in the noise of scattered fluorescence in the TFM image (Fig. 4, Supplementary Fig. 7).

Dendritic spines house the majority of excitatory synapses^43^, and their dynamics can represent critical events in circuit reorganization across development^44-47^ and in response to specific experiences^48-51^. Speed is a major challenge for *in vivo* spine imaging due to the high quality needed for visualizing these small structures (often less than 1 micron in length), and the relatively long dwell times needed to collect sufficient emission photons from dim, thin spines. Due to these speed constraints, many studies rely on a relatively small imaging volume per animal, or perform long anesthesia sessions to reach sufficient sample size^5,44-51^. The PSTPM image shown in Fig. 3J represents a volume of 2,560,000 μm^3^ and took 27 minutes to acquire. The long duration of PSTPM imaging sessions limits these studies to capturing plasticity events that occur between sessions, when animals are awake, often over relatively long timescales. Visualization of spine and synapse dynamic events occurring on a faster time scale is not possible with such slow imaging speeds.

While faster imaging approaches such as traditional TFM have been used for imaging large, bright structures such as neuronal cell bodies^10,11^, they have not been used for imaging subcellular structures because the fluorescence from small, dim structures is lost to scattering. Indeed, in Fig. 3K, we show that in the same 2,560,000 μm^3^ tissue volume imaged in less than 1 minute using TFM, half of the spines are undetectable. However, with our trained DeScatterNet, we were able to recover the vast majority of spines, producing a high-quality output image that matched closely with the ground truth PSTPM data. As an exact comparison of imaging time, to obtain a stack with dimensions of 800 × 800 × 64 pixels, it takes 27.3 minutes for PSTPM, but only 32 seconds for TFM. By overcoming the speed limitation for *in vivo* spine imaging, we can assay spines over fast timescales and with large sample sizes that would be infeasible with current PSTPM approaches. Further, when compared with other conventional approaches, such as a deconvolution algorithm and a denoising neural network, the proposed method provides better correspondence with the ground truth PSTPM data (Supplementary Fig. 4). With the DeScatterNet and forward model, the proposed inversion solver is also able to reconstruct high-quality images from TFM measurements fast with the depth and wavelength parameters integrated.

Thus, we demonstrate that using a deep learning approach combined with TFM, we can image dendritic spines across a large tissue volume more than an order of magnitude faster than with PSTPM. Our approach produces high-quality images without the need for long imaging sessions. Fast, high-quality imaging of large tissue volumes at diffraction limited resolution opens the door to new experimental paradigms in which structural plasticity events can be assayed over shorter timescales and potentially in awake, behaving animals.

## Methods

### 3D Network Architecture:

In this work, a 3D convolutional neural network is used to implement the proposed learning method (Supplementary Figure 1). Similar to the approach presented in^52,53^, in the left path of the neural network, 3D convolutions are performed at each stage to extract the features from the data, and at the end of each stage 3D Max pooling is used for down-sampling purposes. In the right path of the neural network, 3D convolutions are also used to extract the features at each stage, whereas at the end of each stage, transposed 3D convolutions are used to improve the resolution. Both the left and right paths of the network consist of four stages with a connecting middle stage. Each stage includes two to three 3D convolutions coupled with rectified linear unit (ReLU) activation function and batch normalization. Skip connections with concatenations are used between the left and right path to keep the features contained in the left path. Since the inputs and outputs share many important features, a long skip connection is further added to connect the input all the way to the output, forming a residual learning^54^.

In this work, the input data have sizes of 128 × 128 × 64 voxels, and the convolutions performed in the network use volumetric kernels with 3 × 3 × 3 voxels. In the left path, by performing 3D Max pooling at the end of each stage with a pool size of (2, 2, 2), the resulting resolution of feature maps are halved when data proceed through. Meanwhile, due to the number of channels doubled by convolutions at the beginning of each stage, the number of feature maps are doubled. In the right path, the Max pooling is replaced by the transpose 3D convolutions with strides of (2, 2, 2). Thus, when data pass through, the spatial resolution of the feature maps is doubled, and information is gathered to finally output a high-resolution cube with 128 × 128 × 64 voxels.

### Generating Training and Testing Data:

In the training process, a total number of 33 PSTPM image stacks provided ground truth for the training data, which are concatenated as 3D stacks from *in vivo* images acquired from the brains of 26 mice at different depths. Due to inhomogeneity of the brain tissue that also includes blood vessels and meninges, the measured samples are heterogeneous. Before training and testing, we make a threshold (*M*=100) for the maximum value, i.e., all the values larger than *M* are attributed as M. This is important for retaining the dynamic range of the dim features during training. Each PSTPM image stack is interpolated into a dimension of 922 × 922 × 64 pixels. When generating training data, 134 small stacks with a dimension of 128 × 128 × 64 pixels are extracted from each PSTPM image stack, where the positions of these 134 small stacks are randomly distributed in the original PSTPM image stacks. More specifically, the central *x* and *y* indices of these small stacks are randomly selected 134 times from indices that have values larger than *S* (*S*=20) in the large PSTPM image for each *z* depth. Here, *S* is set to make sure that the randomly selected small stacks contain spine features instead of containing only noisy signals. Consequently, there are a total of 134 × 33 = 4422 stacks used as ground truth in the training process, of which 4201 stacks are used as training data and 221 stacks are used as validation data.

In the training process, the inputs of the neural network, i.e., the TFM image stacks *I_TFM_*, are synthetically generated from the PSTPM image stacks *I_PSTPM_*. Specifically, we have *I_TFM,z_*(*x, y*) = *Poiss*[*I_PSTPM,z_*(*x, y*) * *PSF_s,z,λ_*(*x, y*)]. Here, the operators *Poiss*[.] and * represent adding Poisson noise and convolution operations in *x*, *y*, respectively. *PSF_s,z,λ_*(*x, y*) is the scattering point spread function at depth *z* for a fluorophore with a nominal emission wavelength *λ*. *PSF_s,z,λ_*(*x, y*) consists of two components including the ballistic photon distribution and the scattered photon distribution, and is simulated according to the method proposed by Kim et al. ^2^ (Supplementary Section 2).

In this work, the TFM training data is synthetically generated instead of directly using experimental TFM data, which is critical for practical applications: First, generating a sufficiently large training dataset of matching pairs of PSTPM and experimental TFM images would be prohibitively difficult for many labs. The proposed method is therefore more broadly usable when training data from one imaging modality can be generated synthetically. Secondly, even if there is sufficient training data from pairs of experimental TFM and PSTPM images, these images cannot necessarily be aligned on a pixel-by-pixel basis, particularly if the images are acquired on different microscopes. Generating synthetic TFM data enables pixel-by-pixel matching that is critical for training. See Supplementary Section 2 for more detail on this point.

In the testing process, both synthetic data and experimental data are used. First, the synthetic data are generated in the same way as those in the training data, where the input stack is synthetically generated from the experimental PSTPM stack. It is noted that the PSTPM stack used in the test has not been used in the training process. Second, experimental data are used, where the input and output stacks are measured *in vivo* from TFM and PSTPM, respectively. Due to the limited memory of our hardware (Dell with Xeon Silver 4114 CPU, 32GB RAM, NVIDIA TITAN RTX GPU 24 GB), both the inputs and outputs have dimensions of 128 × 128 × 64. Nevertheless, due to the fast speed of the reconstruction, we can reconstruct large dimensional stacks by sticking multiple outputs into a single large stack. For each round of testing, 64 stacks measured from TFM with the size of 128×128×64 pixels for each stack are inputted into the trained network, and the output 64 stacks are finally patched into one large stack with the size of 1024×1024×64. This was done for two separate rounds of experimental data (Figs. 3-4 and Supplementary Figure 6-7). For synthetic results, 16 stacks measured from TFM with the size of 128×128×64 pixels for each stack are inputted into the trained network, and the output 16 stacks are finally patched into one large stack with the size of 512×512×64. We therefore used a total of 64 x 2 = 128 stacks (each measuring 128×128×64 pixels) for testing on experimental data, and a total of 16 stacks (each measuring 128×128×64 pixels) for testing on experimental synthetic data.

### Neural Network Parameters:

Mean squared error is used as loss function in this work. The hyperparameters for the network are as follows: The learning rate is 10^−4^. Adam optimizer is used for optimization. Maximum 30 epochs with 300 steps for each epoch are set for training. The batch size is 3 and channel size is 1. To mitigate the effects of possible overfitting, we empirically apply an “early stopping” strategy. Specifically, the training process is stopped when there is no obvious change in the loss of validation data (Supplementary Figure 2).

### Preparation of *In Vivo* Specimens:

All animal procedures were approved by the Massachusetts Institute of Technology Committee on Animal Care and meet the NIH guidelines for the care and use of vertebrate animals. To enable visualization of individual neurons’ dendritic morphology, *in utero* electroporations were performed on embryonic day 15.5 timed pregnant C57BL/6J mice to express a fluorescent protein in a sparse population of layer 2/3 pyramidal neurons. Constructs used for *in utero* electroporation were a Cre-dependent mScarlet-I cell fill or a Cre-dependent eYFP cell fill^5^ at a concentration of 0.7 *μg/μl*, along with a Cre plasmid^55^ at a concentration of 0.03 *μg/μl*. Fast Green (0.1%) was included in the plasmid solution for visualization. A total of 0.5-1.0 *μl* of the plasmid solution was injected into the right lateral ventricle with a 32 G Hamilton Syringe (Hamilton Company), and five pulses of 36 V (duration 50 ms, frequency 1 Hz) targeting the visual cortex were delivered from a square-wave electroporator (ECM830, Harvard Apparatus). Pups were implanted with a 5 mm cranial window over the right hemisphere as described previously^56^ and fitted with a custom head mount to enable fixation to the microscope stage. All imaging took place under isoflurane anesthesia (1.25%) with the head mount fixed to the microscope stage. The animal whose images are shown in Fig. 3 expressed mScarlet-I as a cell fill, underwent cranial window surgery at postnatal day 14, and was imaged at postnatal day 32. The animal whose images are shown in Supplementary Figure 6 expressed eYFP as a cell fill, underwent cranial window surgery at postnatal day 13, and was imaged at postnatal day 33.

### Collection of PSTPM Images:

*In vivo* PSTPM was performed on a custom-built microscope to visualize the dendritic morphology of layer 2/3 pyramidal neurons in the visual cortex of anesthetized mice. The source of excitation was a Mai Tai HP Ti: Sapphire laser (Rep rate 80 MHz, Spectra Physics) tuned to 1030 nm or 915 nm. The average power delivered to the specimen ranged from 10 to 75 mW depending on cell brightness and imaging depth. Galvanometric XY scanning mirrors (6215H, Cambridge Technology) and a piezo actuator Z positioning system (Piezosystem Jena) were used for XY and Z movement, respectively. The dwell time per pixel was 40 *μs*. This relatively long dwell time was necessary for collecting sufficient emission photons per pixel (~20 photons per pixel) from dendritic spines, which have a weak two-photon cross section per pixel when imaged at diffraction-limited resolution. The pixel size was 0.25 *μm* in XY, and the Z step size was 1 *μm*. The beam was focused by a 20x/1.0 NA water immersion objective lens (W Plan-Apochromat, Zeiss). Emissions were collected by the same objective lens and passed through an IR blocking filter (E700SP, Chroma Technology). Emissions were then separated by dichroic mirrors at 520 nm and 560 nm. After passing through three independent bandpass filters (485/70 nm, 550/100 nm, and 605/75 nm), emissions were collected simultaneously onto three separate PMTs. Raw 2-photon scanning data were processed for spectral linear unmixing and converted into a tif Z stack using Matlab (Mathworks) and ImageJ (NIH). For mice expressing an eYFP cell fill, the 550/100 nm (i.e. yellow) channel was used to visualize the dendritic morphology. For mice expressing an mScarlet-I cell fill, the 605/75 nm (i.e. red) channel was used to visualize the dendritic morphology.

### Collection of TFM Images:

*In vivo* TFM was performed on a custom-built microscope using a 1035 nm fixed wavelength laser (repetition rate 1 MHz, spectrum width ±5 nm, Monaco, Coherent) as the source of excitation. This wavelength adequately excites both eYFP and mScarlet-I, though a shorter wavelength would be optimal for eYFP. The average power delivered to the specimen was 620 mW. The beam was mechanically scanned along the y-axis by a scanning mirror (6350, Cambridge Technology, MA, USA). A cylindrical lens (f=150 mm) focused the beam into a line on a grating (20RG1200-1000-2, Newport Co., CA, USA, 1200 grooves/mm), which generated dispersion along the x-axis. A diffractive optical element (DOE) split the beam into 4 beams in the Fourier plane. After the tube lens and objective lens (XLUMPlanFL, 20x, 0.95NA, Olympus), the diffracted beam focused 4 scanning lines on the imaging plane. The image was detected by 2 sCMOS cameras simultaneously (red channel, Prime95B, Photometrics; yellow channel, PCO edge 5.5, PCO AG). Emissions for the red and yellow channels were separated by a dichroic mirror at 560 nm. Emissions collected in the red channel were used to visualize dendritic morphology labeled by the mScarlet-I cell fill. Emissions collected in the yellow channel were used to visualize dendritic morphology labeled by the eYFP cell fill. The field of view was 250 × 250 μm^2^, the z step size was 1 μm, the pixel size was 170 nm, and the exposure time was 500 ms per plane. We note that the pixel size of the TFM images differed from the pixel size of the PSTPM images. To generate synthetic TFM data, original PSTPM images’ pixel size was changed using bicubic interpolation to match the experimental TFM pixel size. For display purposes, images used for figures in the manuscript were interpolated in ImageJ, and the cropped example images were scaled so that the 2 μm scale bar matched between images.

### Dendritic spine analysis:

Dendritic spines were scored using established criteria based on a consensus of multiple laboratories that perform similar *in vivo* spine analysis^4,5^. Spines were defined as protrusions with a length of at least 0.75 μm (3 pixels in the PSTPM image) from the edge of the dendritic shaft, present in at least two consecutive Z planes. In accordance with consensus methods from multiple *in vivo* imaging groups^4,57^, we only analyze spines that project laterally from the dendrite shaft. These consensus methods are based on the fact that dendritic spines are often less than 1 μm in length, and Z-projecting spines therefore cannot be reliably scored even in PSTPM images due the poorer Z compared to XY resolution.

Spines were first scored on the PSTPM image. Each spine scored in the PSTPM image was then marked as present or absent in the TFM and neural net output images. Spine analysis was performed by two independent human scorers for both the mScarlet- and eYFP-expressing cells, and the results from both scorers are shown for each cell (Fig. 3M, Supplementary Figure 6O).

## Data Availability

The data that support the plots within this paper and other findings of this study are available from the corresponding authors upon reasonable request. The *in vivo* images used for training and testing the neural net are available using the following link (https://doi.org/10.5281/zenodo.6453404).

## Code availability.

The codes that support the plots within this paper and other findings of this study have been uploaded (https://github.com/eleweiz/DeScattering_NN).

## References

[1] Denk W., Strickler J. H. & Webb W. W. Two-photon laser scanning fluorescence microscopy. Science 248, 73 (1990). 2321027 10.1126/science.2321027

[2] Kim K. H. Multifocal multiphoton microscopy based on multianode photomultiplier tubes. Opt. Express 15, 11658–11678 (2007). 19547526 10.1364/oe.15.011658 PMC3060709

[3] Helmchen F. & Denk W. Deep tissue two-photon microscopy. Nature Methods 2, 932–940 (2005). 16299478 10.1038/nmeth818

[4] Holtmaat A. Long-term, high-resolution imaging in the mouse neocortex through a chronic cranial window. Nature Protocols 4, 1128–1144 (2009). 19617885 10.1038/nprot.2009.89 PMC3072839

[5] Chen Jerry L. Clustered Dynamics of Inhibitory Synapses and Dendritic Spines in the Adult Neocortex. Neuron 74, 361–373 (2012). 22542188 10.1016/j.neuron.2012.02.030 PMC3340582

[6] Villa K. L. Inhibitory Synapses Are Repeatedly Assembled and Removed at Persistent Sites In Vivo. Neuron 89, 756–769 (2016). 26853302 10.1016/j.neuron.2016.01.010 PMC4760889

[7] Zhu G., Howe J. v., Durst M., Zipfel W. & Xu C. Simultaneous spatial and temporal focusing of femtosecond pulses. Opt. Express 13, 2153–2159 (2005). 19495103 10.1364/opex.13.002153

[8] Oron D., Tal E. & Silberberg Y. Scanningless depth-resolved microscopy. Opt. Express 13, 1468–1476 (2005). 19495022 10.1364/opex.13.001468

[9] Vaziri A. & Shank C. V. Ultrafast widefield optical sectioning microscopy by multifocal temporal focusing. Opt. Express 18, 19645–19655 (2010). 20940859 10.1364/OE.18.019645

[10] Prevedel R. Fast volumetric calcium imaging across multiple cortical layers using sculpted light. Nature Methods 13, 1021 (2016). 27798612 10.1038/nmeth.4040 PMC5531274

[11] Schrödel T., Prevedel R., Aumayr K., Zimmer M. & Vaziri A. Brain-wide 3D imaging of neuronal activity in Caenorhabditis elegans with sculpted light. Nature Methods 10, 1013 (2013). 24013820 10.1038/nmeth.2637

[12] Belthangady C. & Royer L. A. Applications, promises, and pitfalls of deep learning for fluorescence image reconstruction. Nature Methods (2019). 10.1038/s41592-019-0458-z 31285623

[13] Chen X. D. Computational Methods for Electromagnetic Inverse Scattering. (Wiley, 2018).

[14] Wei Z., Chen R. & Chen X. Nonlinear Reconstruction of Multilayer Media in Scanning Microwave Microscopy. IEEE Transactions on Instrumentation and Measurement 68, 197–205 (2019).

[15] Weigert M. Content-aware image restoration: pushing the limits of fluorescence microscopy. Nature Methods 15, 1090–1097 (2018). 30478326 10.1038/s41592-018-0216-7

[16] Jin K. H., McCann M. T., Froustey E. & Unser M. Deep Convolutional Neural Network for Inverse Problems in Imaging. IEEE Transactions on Image Processing 26, 4509–4522, doi:10.1109/TIP.2017.2713099 (2017). 28641250

[17] Wei Z. & Chen X. Deep-Learning Schemes for Full-Wave Nonlinear Inverse Scattering Problems. IEEE Transactions on Geoscience and Remote Sensing 57, 1849–1860 (2019).

[18] Krizhevsky A., Sutskever I. & Hinton G. E. in Proceedings of the 25th International Conference on Neural Information Processing Systems - *Volume* 1 1097–1105 (Curran Associates Inc., Lake Tahoe, Nevada, 2012).

[19] Ziletti A., Kumar D., Scheffler M. & Ghiringhelli L. M. Insightful classification of crystal structures using deep learning. Nature Communications 9, 2775 (2018). 10.1038/s41467-018-05169-6 PMC6050314 30018362

[20] Nielsen A. A. K. & Voigt C. A. Deep learning to predict the lab-of-origin of engineered DNA. Nature Communications 9, 3135 (2018). 10.1038/s41467-018-05378-z PMC6081423 30087331

[21] Girshick R., Donahue J., Darrell T. & Malik J. in Proceedings of the 2014 IEEE Conference on Computer Vision and Pattern Recognition 580–587 (IEEE Computer Society, 2014).

[22] Ronneberger O., Fischer P. & Brox T. in Medical Image Computing and Computer-Assisted Intervention – MICCAI 2015: 18th International Conference, Munich, Germany, October 5-9, 2015, Proceedings, Part III (eds Navab Nassir, Hornegger Joachim, Wells William M., & Frangi Alejandro F.) 234–241 (Springer International Publishing, 2015).

[23] Eraslan G., Simon L. M., Mircea M., Mueller N. S. & Theis F. J. Single-cell RNA-seq denoising using a deep count autoencoder. Nature Communications 10, 390 (2019). 10.1038/s41467-018-07931-2 PMC6344535 30674886

[24] Eulenberg P. Reconstructing cell cycle and disease progression using deep learning. Nature Communications 8, 463 (2017). 10.1038/s41467-017-00623-3 PMC5587733 28878212

[25] Wei Z. & Chen X. Physics-Inspired Convolutional Neural Network for Solving Full-Wave Inverse Scattering Problems. IEEE Transactions on Antennas and Propagation 67, 6138–6148 (2019).

[26] Zhu B., Liu J. Z., Cauley S. F., Rosen B. R. & Rosen M. S. Image reconstruction by domain-transform manifold learning. Nature 555, 487 (2018). 29565357 10.1038/nature25988

[27] Lee S., Han S., Salama P., Dunn K. W. & Delp E. J. in 2019 IEEE 16th International Symposium on Biomedical Imaging (ISBI 2019). 538–542.

[28] Shajkofci A. & Liebling M. in 2018 25th IEEE International Conference on Image Processing (ICIP). 3818–3822.

[29] Wang H. Deep learning enables cross-modality super-resolution in fluorescence microscopy. Nature Methods 16, 103–110 (2019). 30559434 10.1038/s41592-018-0239-0 PMC7276094

[30] Ouyang W., Aristov A., Lelek M., Hao X. & Zimmer C. Deep learning massively accelerates super-resolution localization microscopy. Nature Biotechnology 36, 460 (2018). 10.1038/nbt.4106 29658943

[31] Nehme E., Weiss L. E., Michaeli T. & Shechtman Y. Deep-STORM: super-resolution single-molecule microscopy by deep learning. Optica 5, 458–464 (2018).

[32] Weigert M., Royer L., Jug F. & Myers G. in Medical Image Computing and Computer-Assisted Intervention – MICCAI 2017. 126–134 (Springer International Publishing).

[33] Rivenson Y. Deep learning microscopy. Optica 4, 1437–1443 (2017).

[34] Wang R. & Tao D. Non-Local Auto-Encoder With Collaborative Stabilization for Image Restoration. IEEE Transactions on Image Processing 25, 2117–2129 (2016). 26978824 10.1109/TIP.2016.2541318

[35] Wei Z. & Chen X. Induced-Current Learning Method for Nonlinear Reconstructions in Electrical Impedance Tomography. IEEE Transactions on Medical Imaging, doi:10.1109/TMI.2019.2948909 (2019). 31647424

[36] McCann M. T., Jin K. H. & Unser M. Convolutional Neural Networks for Inverse Problems in Imaging: A Review. IEEE Signal Processing Magazine 34, 85–95 (2017).

[37] Xue Y. Scattering reduction by structured light illumination in line-scanning temporal focusing microscopy. Biomed. Opt. Express 9, 5654–5666 (2018). 30460153 10.1364/BOE.9.005654 PMC6238912

[38] Yi Xue J. R. B., Wadduwage Dushan N., Park Jong Kang, Nedivi Elly, So Peter T. C.. Multiline Orthogonal Scanning Temporal Focusing (mosTF) Microscopy for Scattering Reduction in High-speed in vivo Brain Imaging. arXiv preprint arXiv:1905.11540 (2019). 10.1038/s41598-024-57208-6 PMC11091065 38740797

[39] Escobet-Montalbán A. Wide-field multiphoton imaging through scattering media without correction. Science Advances 4, eaau1338 (2018). 30333995 10.1126/sciadv.aau1338 PMC6184782

[40] Wadduwage D. N., Park J. K., Boivin J. R., Xue Y. & So P. T. De-scattering with Excitation Patterning (DEEP) Enables Rapid Wide-field Imaging Through Scattering Media. arXiv preprint arXiv:1902.10737 (2019). 10.1126/sciadv.aay5496 PMC8262816 34233883

[41] Hansen P. C., Nagy J. G. & O'Leary D. P. Deblurring Images: Matrices, Spectra, and Filtering. (Society for Industrial and Applied Mathematics, 2006).

[42] Wallace W., Schaefer L. H. & Swedlow J. R. A Workingperson’s Guide to Deconvolution in Light Microscopy. BioTechniques 31, 1076–1097, doi:10.2144/01315bi01 (2001). 11730015

[43] DeFelipe J. & Fariñas I. The pyramidal neuron of the cerebral cortex: Morphological and chemical characteristics of the synaptic inputs. Progress in Neurobiology 39, 563–607 (1992). 1410442 10.1016/0301-0082(92)90015-7

[44] Boivin J. R., Piekarski D. J., Thomas A. W. & Wilbrecht L. Adolescent pruning and stabilization of dendritic spines on cortical layer 5 pyramidal neurons do not depend on gonadal hormones. Developmental Cognitive Neuroscience 30, 100–107 (2018). 29413532 10.1016/j.dcn.2018.01.007 PMC6294327

[45] Holtmaat A. J. G. D. Transient and Persistent Dendritic Spines in the Neocortex In Vivo. Neuron 45, 279–291 (2005). 15664179 10.1016/j.neuron.2005.01.003

[46] Johnson C. M. Long-range orbitofrontal and amygdala axons show divergent patterns of maturation in the frontal cortex across adolescence. Developmental Cognitive Neuroscience 18, 113–120 (2016). 26896859 10.1016/j.dcn.2016.01.005 PMC5283395

[47] Zuo Y., Lin A., Chang P. & Gan W.-B. Development of Long-Term Dendritic Spine Stability in Diverse Regions of Cerebral Cortex. Neuron 46, 181–189 (2005). 15848798 10.1016/j.neuron.2005.04.001

[48] Fu M., Yu X., Lu J. & Zuo Y. Repetitive motor learning induces coordinated formation of clustered dendritic spines in vivo. Nature 483, 92–95 (2012). 22343892 10.1038/nature10844 PMC3292711

[49] Muñoz-Cuevas F. J., Athilingam J., Piscopo D. & Wilbrecht L. Cocaine-induced structural plasticity in frontal cortex correlates with conditioned place preference. Nature Neuroscience 16, 1367 (2013). 23974707 10.1038/nn.3498 PMC3940437

[50] Xu T. Rapid formation and selective stabilization of synapses for enduring motor memories. Nature 462, 915–919 (2009). 19946267 10.1038/nature08389 PMC2844762

[51] Yang G., Pan F. & Gan W.-B. Stably maintained dendritic spines are associated with lifelong memories. Nature 462, 920–924 (2009). 19946265 10.1038/nature08577 PMC4724802

[52] Milletari F., Navab N. & Ahmadi S. in 2016 Fourth International Conference on 3D Vision (3DV). 565–571.

[53] Çiçek Ö., Abdulkadir A., Lienkamp S. S., Brox T. & Ronneberger O. in Medical Image Computing and Computer-Assisted Intervention – MICCAI 2016. 424–432 (Springer International Publishing).

[54] He K., Zhang X., Ren S. & Sun J. in 2016 IEEE Conference on Computer Vision and Pattern Recognition (CVPR). 770–778.

[55] Subramanian J., Dye L. & Morozov A. Rap1 Signaling Prevents L-Type Calcium Channel-Dependent Neurotransmitter Release. The Journal of Neuroscience 33, 7245 (2013). 23616533 10.1523/JNEUROSCI.5963-11.2013 PMC3657727

[56] Lee W.-C. A. Dynamic Remodeling of Dendritic Arbors in GABAergic Interneurons of Adult Visual Cortex. PLOS Biology 4, e29 (2005). 16366735 10.1371/journal.pbio.0040029 PMC1318477

[57] Holtmaat A. Long-term, high-resolution imaging in the mouse neocortex through a chronic cranial window. Nature protocols 4, 1128–1144, doi:10.1038/nprot.2009.89 (2009). 19617885 PMC3072839

